# Supplementary material for: Photo-switchable tweezers illuminate pore-opening motions of an ATP-gated P2X ion channel
Source: eLife. 2016 Jan 25;5:e11050. doi: 10.7554/eLife.11050 (PMC4739762; doi:10.7554/eLife.11050)
Supplement: Figure 1—source data 1. — DOI: http://dx.doi.org/10.7554/eLife.11050.004 [file elife-11050-fig1-data1.docx]

**Figure 1—source data 1.** Interatomic distances between pairwise residues

| Pairwise residues | Relative distances in apo state (Å) | Relative distances in the ATP-bound state (Å) |
| --- | --- | --- |
| Y47… Y47 | 26.6 ± 0.1 | 28.3 ± 0.5 |
| V48 … V48 | 18.6 ± 0.2 | 22.8 ± 0.4 |
| Q52 … Q52 | 20.9 ± 0.1 | 24.0 ± 0.2 |
| D57 … D57 | 15.1 ± 0.0 | 26.6 ± 0.3 |
| S58 … S58 | 13.3 ± 0.1 | 20.0 ± 0.2 |
| S326 … S326 | 19.9 ± 0.1 | 31.0 ± 0.2 |
| I328 … I328 | 16.1 ± 0.1 | 27.6 ± 0.4 |
| P329 … P329 | 12.0 ± 0.2 | 23.4 ± 0.4 |
| I332 … I332 | 7.2 ± 0.2 | 19.4 ± 0.2 |
| N333 … N333 | 11.3 ± 0.3 | 20.8 ± 0.2 |
|  |  |  |
| I328 … V343 | 15.6 ± 0.2 | 18.9 ± 0.2 |
| I328 … G344 | 16.0 ± 0.2 | 18.5 ± 0.2 |
| I328 … S345 | 20.5 ± 0.2 | 23.2 ± 0.2 |
| I328 … F346 | 20.9 ± 0.1 | 23.1 ± 0.3 |
| I328 … L347 | 17.5 ± 0.2 | 18.8 ± 0.3 |
| I328 … C348 | 19.9 ± 0.4 | 22.2 ± 0.3 |
| I328 … D349 | 24.4 ± 0.3 | 25.9 ± 0.3 |
| I328 … W350 | 23.9 ± 0.2 | 22.2 ± 0.3 |

Values (means ± s.e.m.) are the average distances separating the side chain β-atoms (Cβ -Cβ) of indicated residues from all three subunits of P2X2 homology models built from X-ray structures of the zfP2X4 receptor (Hattori & Gouaux, 2012). For G344, the Cα was selected.
