# Supplementary material for: Photo-switchable tweezers illuminate pore-opening motions of an ATP-gated P2X ion channel
Source: eLife. 2016 Jan 25;5:e11050. doi: 10.7554/eLife.11050 (PMC4739762; doi:10.7554/eLife.11050)
Supplement: Figure 1—source data 2. — DOI: http://dx.doi.org/10.7554/eLife.11050.005 [file elife-11050-fig1-data2.docx]

**Figure 1—source data 2.** Estimated EC_50_ and Hill coefficients for ATP activation

| Constructs | ATP EC_50_ (μM) | *n*_H_ |
| --- | --- | --- |
|  |  |  |
| rP2X2-3T | 19.3 ± 2.6 | 1.4 ± 0.1 |
| Y47C^a^ | 2.5 ± 0.9 | 1.1 ± 0.1 |
| V48C^a^ | 9.4 ± 4.7 | 1.6 ± 0.0 |
| Q52C^a^ | 93.9 ± 13.8 | 1.7 ± 0.1 |
| D57C | 15.2 ± 2.9 | 1.2 ± 0.1 |
| S58C | 18.3 ± 1.4 | 1.5 ± 0.2 |
| S326C | 16.5 ± 3.4 | 1.3 ± 0.2 |
| I328C^a^ | 5.1 ± 0.9 | 1.3 ± 0.1 |
| P329C^a^ | 7. ± 1.0 | 0.9 ± 0.1 |
| I332C^a^ | 9.6 ± 3.0 | 1.0 ± 0.1 |
| N333C^a^ | 9.2 ± 4.2 | 2.2 ± 1.0 |
| V343C | 32.3 ± 9.0 | 1.1 ± 0.1 |
| G344C | 73.5 ± 10.7 | 1.2 ± 0.1 |
| S345C | 17.2 ± 2.2 | 1.1 ± 0.1 |
| F346C | 18.7 ± 3.1 | 1.9 ± 0.3 |
| L347C | 44.6 ± 9.4 | 1.4 ± 0.1 |
| T348C | 27.3 ± 7.8 | 1.4 ± 0.1 |
| D349C^b^ | N.D. | N.D. |
| W350C | 54.1 ± 8.6 | 1.5 ± 0.1 |
| V343C/I328C | 8.5 ± 1.3 | 1.2 ± 0.2 |
| G344C/I328C | 18.2 ± 3.4 | 1.0 ± 0.0 |
| S345C/I328C | 6.7 ± 0.6 | 1.5 ± 0.2 |
| S345C/I328S | 10.6 ± 3.3 | 1.1 ± 0.2 |
| F346C/I328C | 7.7 ± 2.1 | 1.3 ± 0.1 |
| L347C/I328C | 12.4 ± 3.2 | 1.4 ± 0.1 |
| T348C/I328C | 8.7 ± 3.0 | 1.2 ± 0.1 |
| D349C/I328C | N.F. | N.F. |
| W350C/I328C | 21.5 ± 4.2 | 1.2 ± 0.1 |
|  |  |  |
|  |  |  |
| I328C at 365nm | 3.8 ± 0.5 | 1.2 ± 0.2 |
| I328C at 525nm | 3.3 ± 0.4 | 1.4 ± 0.2 |
| I328C/S345C at 365nm | 0.5 ± 0.1 | 0.5 ± 0.1 |
| I328C/S345C at 525nm | 1.9 ± 0.3 | 0.6 ± 0.1 |
|  |  |  |
|  |  |  |
| rP2X2 WT | 39.6 ± 7.0 | 1.5 ± 0.1 |
| G342P in rP2X2 | 1.6 ± 0.4 | 1.1 ± 0.1 |
| G342A inrP2X2 | 94.1 ± 13.0 | 1.3 ± 0.1 |
|  |  |  |

All cysteine mutants were generated on the rP2X2-3T background. G342P and G342A mutations were generated on the rP2X2 receptor. All data are means ± s.e.m., n = 4-7 from at least two transfections. Concentration-response relationships for activation by ATP at 365 nm and 525 nm were determined in cells previously treated for 20 min with 1 μM (for I328C/S345C) or 50 μM MAM (for I328C) in the presence of 3 μM ATP. For I328C, ATP-gated currents were measured at 350 ms under 525 nm light or in the dark after illumination at 365 nm. For I328C/S45C, ATP-gated currents were measured in the dark after switching to the indicated wavelength. N.F. not functional; N.D. not determined. ^a^Data taken from (Lemoine et al, 2013). ^b^Because currents were unstable, EC_50_ and *n*_H_ cannot be determined.
