# Supplementary material for: Photo-switchable tweezers illuminate pore-opening motions of an ATP-gated P2X ion channel
Source: eLife. 2016 Jan 25;5:e11050. doi: 10.7554/eLife.11050 (PMC4739762; doi:10.7554/eLife.11050)
Supplement: Figure 2—source data 1. — DOI: http://dx.doi.org/10.7554/eLife.11050.014 [file elife-11050-fig2-data1.docx]

**Figure 2—source data 1.** Relative ion permeability for chloride

| Constructs | *E*_rev_ NaCl (mV) | *E*_rev_ Man (mV) | *E*_rev_ Na-Ise (mV) | *P*_Cl_/*P*_Na_ |
| --- | --- | --- | --- | --- |
| ATP-gated  P2X2-3T | -4.3 ± 5.4^a^ | -32.5 ± 9.4^a^ | 1.2 ± 5.9^a^ | 0.10 ± 0.14^a^ |
| Light-gated  I328C | 1.2 ± 1.7 | -25.0 ± 2.5 | -2.8 ± 2.1 | 0.09 ± 0.03 |
| I328C/S345C | -2.8 ± 1.9 | -24.7 ± 2.8 | -1.7 ± 1.9 | 0.14 ± 0.03 |

Data are means ± s.e.m., n = 8-9 from at least two transfections. For the P2X2-3T receptor, ATP concentration was 10 μM. Labeling of mutants was performed in the presence of 3 μM ATP and 1 μM (for I328C/S345C) or 50 μM (for I328C) MAM. ^a^Data taken from (Lemoine et al, 2013).
