# Supplementary material for: Photo-switchable tweezers illuminate pore-opening motions of an ATP-gated P2X ion channel
Source: eLife. 2016 Jan 25;5:e11050. doi: 10.7554/eLife.11050 (PMC4739762; doi:10.7554/eLife.11050)
Supplement: Figure 2—source data 2. — DOI: http://dx.doi.org/10.7554/eLife.11050.015 [file elife-11050-fig2-data2.docx]

**Figure 2—source data 2.** Relative ion permeability for calcium

| Constructs | *E*_rev_ NaCl (mV) | *E*_rev_ Ca (mV) | *P*_Ca_/*P*_Na_ |
| --- | --- | --- | --- |
| ATP-gated  P2X2-3T | 7.7 ± 3.6^a^ | 9.2 ± 2.8^a^ | 2.08 ± 0.22^a^ |
| Light-gated  I328C | 1.1 ± 2.5 | -5.3 ± 2.7 | 1.30 ± 0.04 |
| I328C/S345C | -0.4 ± 2.2 | -4.4 ± 1.5 | 1.53 ± 0.14 |

Data are means ± s.e.m., n = 8 from at least two transfections. For the P2X2-3T receptor, ATP concentration was 10 μM in NaCl solutions and 300 μM in Ca solutions. Labeling of mutants was performed in the presence of 3 μM ATP and 1 μM (for I328C/S345C) or 50 μM (for I328C) MAM. ^a^Data taken from (Lemoine et al, 2013).
