# Supplementary material for: Photo-switchable tweezers illuminate pore-opening motions of an ATP-gated P2X ion channel
Source: eLife. 2016 Jan 25;5:e11050. doi: 10.7554/eLife.11050 (PMC4739762; doi:10.7554/eLife.11050)
Supplement: Figure 2—source data 3. — DOI: http://dx.doi.org/10.7554/eLife.11050.016 [file elife-11050-fig2-data3.docx]

**Figure 2—source data 3.** Relative ion permeability for NMDG

|  |  |  | *E*_rev_ NMDG (mV) | |  | *P*_NMDG_/*P*_Cs_ | |
| --- | --- | --- | --- | --- | --- | --- | --- |
| Constructs | *E*_rev_ NaCl (mV) |  | initial | 60s |  | initial | 60s |
| ATP-gated  P2X2-3T | -9.8 ± 5.7^a^ |  | -73.7 ± 5.5^a^ | -49.1 ± 10.5^a^ |  | 0.07 ± 0.01^a^ | 0.20 ± 0.06^a^ |
| Light-gated  I328C | -3.2 ± 1.4 |  | -78.3 ± 1.8 |  |  | 0.04 ± 0.01 |  |
| I328C/S345C | -4.9 ± 1.3 |  | -49.3 ± 2.5 |  |  | 0.15 ± 0.01 |  |

Data are means ± s.e.m., n = 6-9 from at least two transfections. For the P2X2-3T receptor, ATP concentration was 100 μM. Labeling of mutants was performed in the presence of 3 μM ATP and 1 μM (for I328C/S345C) or 50 μM (for I328C) MAM. ^a^Data taken from (Lemoine et al, 2013).
