# Supplementary material for: Photo-switchable tweezers illuminate pore-opening motions of an ATP-gated P2X ion channel
Source: eLife. 2016 Jan 25;5:e11050. doi: 10.7554/eLife.11050 (PMC4739762; doi:10.7554/eLife.11050)
Supplement: Figure 2—source data 4. — DOI: http://dx.doi.org/10.7554/eLife.11050.017 [file elife-11050-fig2-data4.docx]

**Figure 2—source data 4.** Single-channel properties of light-gated and ATP-gated receptors

|  |  | Conductance (pS) | | | |  | |  | | Relative area (%) | | | | |  |
| --- | --- | --- | --- | --- | --- | --- | --- | --- | --- | --- | --- | --- | --- | --- | --- |
|  | Full open state |  | Sub-states | | | |  | | Full open state | |  | Sub-states | | | |
| Constructs | O |  | S | S1 | S2 | |  | | O | |  | S | S1 | S2 | |
| ATP-gated  rP2X2-3T | 32.2 ± 0.7 |  | 15.0 ± 1.3 | - | - | |  | | 11.1 ± 3.6 | |  | 9.5 ± 2.4 | - | - | |
| Light-gated  I328C | 20.4 ± 1.4 |  | 9.8 ± 2.0 | - | - | |  | | 5.8 ± 1.4 | |  | 11.9 ± 0. 7 | - | - | |
| I328C/S345C | 24.9 ± 0.7 |  | - | 5.4 ± 0.5 | 13.6 ± 0.8 | |  | | 6.2 ± 0.4 | |  | - | 23.5 ± 4.9 | 14.9 ± 4.6 | |

Data are means ± s.e.m. (n = 4-6 patches)
